# SysMod: the ISCB community for data-driven computational modelling and multi-scale analysis of biological systems

**DOI:** 10.1093/bioinformatics/btab229

**Published:** 2021-06-24

**Authors:** Andreas Dräger, Tomáš Helikar, Matteo Barberis, Marc Birtwistle, Laurence Calzone, Claudine Chaouiya, Jan Hasenauer, Jonathan R Karr, Anna Niarakis, María Rodríguez Martínez, Julio Saez-Rodriguez, Juilee Thakar

**Affiliations:** Computational Systems Biology of Infections and Antimicrobial-Resistant Pathogens, Institute for Bioinformatics and Medical Informatics (IBMI), University of Tübingen, 72076 Tübingen, Germany; Department of Computer Science, University of Tübingen, 72076 Tübingen, Germany; German Center for Infection Research (DZIF), Partner Site, Tübingen, Germany; Cluster of Excellence ‘Controlling Microbes to Fight Infections’, University of Tübingen, Tübingen, Germany; Department of Biochemistry, University of Nebraska, Lincoln, NE 68588-0664, USA; Systems Biology, School of Biosciences and Medicine, Faculty of Health and Medical Sciences, University of Surrey, Guildford GU2 7XH, Surrey, UK; Centre for Mathematical and Computational Biology, CMCB, University of Surrey, Guildford GU2 7XH, Surrey, UK; Synthetic Systems Biology and Nuclear Organization, Swammerdam Institute for Life Sciences, University of Amsterdam, 1098 XH Amsterdam, The Netherlands; Department of Chemical and Biomolecular Engineering, Clemson University, Clemson, SC 29634, USA; Institut Curie, PSL Research University, Mines Paris Tech, Inserm, U900, F-75005 Paris, France; Aix-Marseille Université, CNRS, Centrale Marseille, I2M, Marseille 2780-156, France; Instituto Gulbenkian de Ciência, Oeiras 2780-156, Portugal; Interdisicplinary Research Unit Mathematics and Life Sciences, University of Bonn, Bonn 53115, Germany; Department of Genetics & Genomic Sciences, Icahn School of Medicine at Mount Sinai, New York, NY 10029, USA; GenHotel, University of Evry, University of Paris-Saclay, Genopole, Évry 91025, France; Lifeware Group, Inria Saclay-île de France, 91120 Palaiseau, France; IBM Research Europe, Zurich, CH–8803 Rüschlikon, Switzerland; Heidelberg University, Faculty of Medicine and Heidelberg University Hospital, Institute of Computational Biomedicine, 69120 Heidelberg, Germany; Department of Microbiology and Immunology, University of Rochester School of Medicine and Dentistry, Rochester, NY 14642, USA; Department of Biostatistics and Computational Biology, University of Rochester School of Medicine and Dentistry, Rochester, NY 14642, USA

## Abstract

Computational models of biological systems can exploit a broad range of rapidly developing approaches, including novel experimental approaches, bioinformatics data analysis, emerging modelling paradigms, data standards and algorithms. A discussion about the most recent advances among experts from various domains is crucial to foster data-driven computational modelling and its growing use in assessing and predicting the behaviour of biological systems. Intending to encourage the development of tools, approaches and predictive models, and to deepen our understanding of biological systems, the Community of Special Interest (COSI) was launched in Computational Modelling of Biological Systems (SysMod) in 2016. SysMod’s main activity is an annual meeting at the Intelligent Systems for Molecular Biology (ISMB) conference, which brings together computer scientists, biologists, mathematicians, engineers, computational and systems biologists. In the five years since its inception, SysMod has evolved into a dynamic and expanding community, as the increasing number of contributions and participants illustrate. SysMod maintains several online resources to facilitate interaction among the community members, including an online forum, a calendar of relevant meetings and a YouTube channel with talks and lectures of interest for the modelling community. For more than half a decade, the growing interest in computational systems modelling and multi-scale data integration has inspired and supported the SysMod community. Its members get progressively more involved and actively contribute to the annual COSI meeting and several related community workshops and meetings, focusing on specific topics, including particular techniques for computational modelling or standardisation efforts.

## 1 Introduction

Advances in experimental technologies, such as multi-omics, sequencing and mass spectrometry, among others, have resulted in unprecedented amounts of highly heterogeneous data. Computational modelling and methods enable integrating these data into systems models representing the underlying complexity and, typically, networks of interconnected biological processes. Novel methods and the types of -omics data being integrated into these models (whether to calibrate, parameterise or validate them) are vast. They may, for instance, include transcriptomics, proteomics and metabolomics, at the bulk and single-cell levels. Simulations of these models, in turn, allow us to interrogate the models’ dynamics, characterise their emergent properties, and predict new properties (e.g. drug targets, biomarkers).

Computational modelling also plays an essential role in precision medicine. In this field, dynamic models are, for instance, used to model an individual patient’s disease trajectory, facilitating patient stratification and patient-specific predictions (e.g. of drug responses). Computational modelling continues to push the boundaries by expanding to whole-cell, multi-scale and multicellular models, providing new opportunities to accelerate the advancements of fundamental and applied sciences, such as molecular biology, bioengineering and medicine. Interdisciplinary collaborations among computational systems biologists, molecular biologists, biochemists, engineers, biotechnologists, computer scientists and mathematicians will continue to be critical to leverage the potential of the field altogether.

**Fig. 1.**
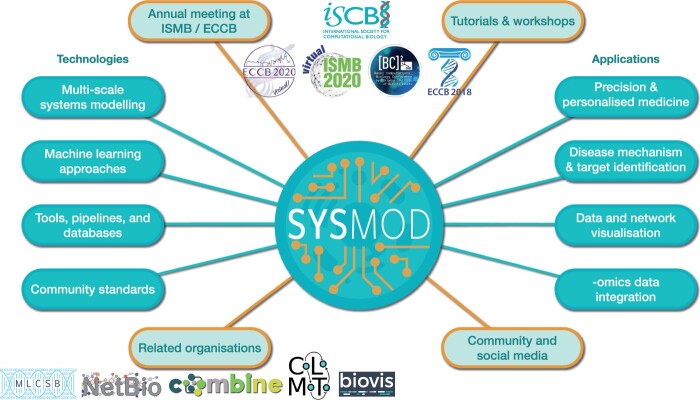
Overview of the COSI SysMod. SysMod is an organisation within the ISCB that offers a variety of activities to its community, such as tutorials, workshops and annual meetings as part of the conferences ISMB/ECCB. To this end, it provides various channels for communication, including popular social media platforms and mailing lists. SysMod connects researchers from diverse fields whose expertise covers a plethora of topics on technologies and their applications. Members of the SysMod community are active in related organisations, such as those shown to the bottom left.

In 2016, the SysMod Community of Special Interest (COSI) of the International Society for Computational Biology (ISCB) was established to foster communication among systems biologists, computational biologists and bioinformaticians. Figure 1 depicts SysMod’s activities, topic areas and integration into an ecosystem within the ISCB by thematic COSIs focusing on complementary yet adjacent subjects. The thematically closely related COSIs include Network Biology (NetBio), Biological Data Visualisation (BioVis), and, since 2018, Machine Learning in Computational Systems Biology (MLCSB). SysMod envisions to provide an interdisciplinary forum for discussing latest developments towards predictive, knowledge-based computer models of biological systems and processes (such as dynamic or logical models of metabolism, signalling and regulatory processes) as well as methods that advance our understanding in biological science, precision medicine and synthetic biology. By fostering interdisciplinary collaborations and applying approaches from different fields that combine modelling and data, SysMod enables an advance in technology, data analysis and data sharing as an opportunity to use data to create more accurate, detailed and re-usable predictive models.

## 2 Resources

SysMod invites researchers to contribute, contact and interact with others in a variety of ways beyond its annual meetings. The SysMod Twitter account https://twitter.com/cosi_sysmod, the YouTube channel https://www.youtube.com/channel/UCcbfopn_ egkcPKw3oBrtnJg, the mailing list at mailto:@sysmod@googlegroups.com and the SysMod website (https://sysmod.info) contain information about the most recent events relevant to the community.

## 3 Annual meetings

### 3.1 First annual meeting: Orlando, 2016

The first SysMod event was a one-day meeting on July 9, 2016, before the ISMB Conference in Orlando, Florida. The meeting featured a poster session, eight contributed talks and three keynote presentations by (i) Vassily Hatzimanikatis, École Polytechnique Fédérale de Lausanne (EPFL), ‘Towards kinetic modelling of genome-scale metabolic networks without sacrificing stoichiometric, thermodynamic and physiological constraints’ (Chakrabarti *et al.*, 2013), (ii) Nathan Price, Institute for Systems Biology (ISB) and the University of Washington, ‘Metabolic analyses in microbes and humans’ (Imam *et al.*, 2015) and (iii) Ioannis Xenarios, Swiss Institute of Bioinformatics (SIB), ‘From biocuration to model predictions and back’. In a joint session with the NetBio COSI, four additional presentations followed the next day.

### 3.2 Second annual meeting: Prague, 2017

The second annual SysMod meeting took place on July 22, 2017, during the European Conference on Computational Biology (ECCB)/ISMB in Prague, Czech Republic. A common theme spanning the three sessions was variability resulting from biological systems, experiments and simulations. The talks were organised into three thematic sessions: (i) Accounting for uncertainty in modelling and simulation, (ii) Using modelling to understand gene regulation, signalling and response to drugs, (iii) Genome-scale and multi-scale modelling. Two keynotes were delivered by (i) Rainer Breitling, University of Manchester, ‘Modelling in uncertain times,’ and (ii) Jana Wolf, Max Delbrück Center for Molecular Medicine, ‘Computational modelling of promoter occupancies in MYC-dependent gene regulation’ (Lorenzin *et al*., 2016). The 13 speakers discussed how to maximise the accuracy and usefulness of computational models, given existing challenges. In 2017, SysMod accepted, for the first time, four proceedings papers (Cinquemani *et al.*, 2017; Damiani *et al.*, 2017; Kazeroonian *et al.*, 2017; Pischel *et al.*, 2017) out of 37 initial submissions.

### 3.3 Third annual meeting: Chicago, 2018

The third annual SysMod meeting took place on July 7, 2018, during the ISMB in Chicago, Illinois, and featured three sessions: (i) ‘Detailed models of individual cellular processes,’ (ii) ‘Comprehensive models of cells and tissues,’ and (iii) ‘Models of human physiology and disease’. Keynote presentations were delivered by (i) Andre Levchenko, Yale University, ‘Variability and phenotype selection in invasive cancer spread,’ (ii) Peter Sorger, Harvard Medical School, ‘Trials, biomarkers, and data science,’ and (iii) John Tyson, Virginia Tech, ‘The cell division cycle: a closed loop of switches embedded in switches’ (Novak *et al.*, 2018). The meeting also featured ten contributed talks, four lightning talks and 33 posters. A recurrent theme was the need for multi-scale models to integrate emergent molecular behaviours and patterns into processes and data at multiple scales of biological organisation. Several presenters discussed how signal integration across different scales was critical to define the interactions among cellular processes, bacterial infection and cancer spread. The meeting stimulated vibrant discussions on challenges in systems modelling and the need for collaboration within the modelling community, particularly regarding the development of comprehensive multi-scale models. This discussion called for further efforts to make modelling more modular, improve the association of models with their underlying data and assumptions, and verify models, thus rigorously improving accuracy.

### 3.4 Fourth annual meeting: Basel, 2019

The fourth annual SysMod meeting took place on July 22, 2019, during the ISMB in Basel, Switzerland, and comprised three sessions spanning human cells and disease modelling, systems biology of microorganisms, and current trends in the field. Each of the three sessions featured a keynote speaker: (i) Trey Ideker, University of California, San Diego, ‘Interpreting the cancer genome through physical and functional models of the cancer cell,’ (ii) Edda Klipp, Humboldt-Universität zu Berlin, Germany, ‘Systematic integration of models and data for yeast growth, division and stress response’ (Münzner *et al.*, 2019) and (iii) Jörg Stelling, ETH Zürich, Switzerland ‘Systems analysis of cell-to-cell variability’ (Azizoglu and Stelling, 2019). The first session, ‘Systems biology of human cells and diseases,’ covered the following themes: modelling approaches for personalised medicine, disease diagnosis and treatment, and approaches to investigate heterogeneity in healthy and diseased tissues, such as tumours or in immune responses. The second session, ‘Systems biology of microorganisms,’ was centred around the use of -omics data for integrative modelling of processes that are strongly interlinked at the cellular level but measured separately at the population and single-cell levels. Further topics included computational tools to analyse -omics data and to predict metabolic interactions within microbial communities. The final session concluded the meeting with presentations on ‘Current trends in systems biology.’ These presentations discussed various computational approaches to infer dynamical information such as protein-folding states, mutation pathways and rates from an ensemble of instantaneous data, i.e. one time-point data. Physical whole-cell models that can connect molecular structure with biological function were also discussed. Such models aim at predicting how changes at the molecular level propagate to alter function at the cellular level. Furthermore, these models can improve our understanding of how biological macromolecules behave inside cells while navigating a spectrum of specific and non-specific interactions in the presence of a variety of electrolytes, osmolytes and other small molecules. Further discussion included how modelling techniques, combined with large-scale -omics data, facilitate the elucidation of biochemical regulation mechanisms.

### 3.5 Fifth annual meeting: ‘Virtual’, 2020

Due to the COVID-19 pandemic, the fifth annual SysMod meeting was held on July 13 and 14, 2020, as a virtual conference. The meeting featured three keynote speakers: (i) Paul François, McGill University, delivered the first keynote presentation, ‘Robotic mapping and generative modelling of cytokine responses.’ In this talk, he described an evolutionary algorithm to identify features of antigenicity using high-throughput temporal cytokine profiling. (ii) Douglas Lauffenburger, Massachusetts Institute of Technology as the second keynote speaker, presented ‘Cross-species translation of biological information via computational systems modelling frameworks’ (Brubaker *et al.*, 2019), in which he outlined a systems biology and machine learning approach to translate genetic and phenotypic information from animal models to humans for pre-clinical to clinical stages. Finally, (iii) Carolyn Cho, Merck, Inc., gave the third keynote presentation on the topic ‘Quantitative and Systems Pharmacology (QSP) and Model-Informed Drug Development (MIDD) of a ‘Smart’ Insulin,’ which can evaluate therapeutic modulation of its target and assist human dose prediction as well as the determination of its efficacy (Visser *et al.*, 2020).

This fifth SysMod meeting in 2020 discussed various modelling techniques, including flux balance analysis, machine learning, logical and kinetic modelling, combined with their application to a multitude of biological systems. The contributed presentations focused on constraint-based modelling of metabolic networks, organism-level modelling, novel methods for network modelling and modelling of cell populations. In the first session, metabolic models were developed to rationalise strategies for (i) the optimisation of energy use, such as to identify alternative sources of reactive oxygen species, (ii) to optimise proteomic allocation, integrate different sources of information and (iii) to identify a reduced genome for functional optimisation (Szeliova *et al.*, 2020). The second session on organism-level models included presentations on (i) a scalable model for prokaryote and human cells using a modular pipeline, (ii) a cross-inhibited Turing reaction-diffusion system to model the regeneration and homeostasis in planaria (Herath and Lobo, 2020) and (iii) the first mathematical model of cell cycle control in budding yeast that can exhibit sustained, autonomous oscillations through previously unknown network designs (Mondeel *et al.*, 2020). These talks described the role of modelling in revealing underlying mechanistic insights by predicting novel molecular regulations.

Further presentations described how to infer missing information on kinase-substrate interactions using an electric circuit-based model for network propagation and how loopy belief propagation with node belief updates can be used to investigate gene probability of being in specific states (Kotiang and Eslami, 2020). In the final session, studies using mixed cell populations in tissue and Peripheral Blood Mononuclear Cells (PBMCs) were presented. Single-cell RNA sequencing data of PBMCs was analysed using pathway activities measured by Boolean Omics Network Invariant-Time Analysis (BONITA, Palli *et al.*, 2019). Also, sorting, intercalation, and involution of tissue behaviours due to regulated cell adhesion were investigated using partial differential equations (Ko and Lobo, 2019). During the second day, a stochastic spatio-temporal model of DNA replication that incorporates experimental genome structural data and protein mobility dynamics and probabilistic activation firing was presented (Windhager *et al.*, 2019). The final talk gave a metric to exploit concepts of reliability engineering, elementary flux modes, and minimal cut sets to calculate the robustness of *E.coli*, *Shigella*, *Salmonella* and fungal genome-scale metabolic models (Libiseller-Egger *et al.*, 2020). In this context, the amino-acid metabolism was stated to be more fragile than the central carbon metabolism. Throughout the virtual SysMod meeting, PhD students and postdocs lively discussed efforts to integrate further -omics data to improve mechanistic understanding of biological processes.

## 4 Highlights and outcomes

**Fig. 2.**
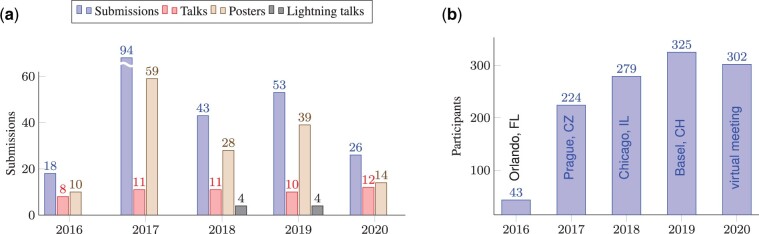
Contributions and attendance of the SysMod annual meetings. (**a**) Numbers of keynotes, talks, lightning talks and posters contributed to the SysMod meetings. In 2018, the inception of the COSI for Machine Learning in Computational Systems Biology (MLCSB) enabled authors to submit their works to communities with specialised focus areas. (**b**) Numbers of registered conference participants of the SysMod meetings from 2016 to 2020.

The various SysMod meetings had a substantial impact, and the community met with great interest (Fig. 2). The formats of scientific contributions varied (Fig. 2a), and the participation numbers steadily increased in the first four years while remaining almost constant during COVID-19 (Fig. 2b). High-quality contributions from the community and keynote speakers from world-renowned institutes and the pharmaceutical industry, have undoubtedly helped fuel this success.

Since 2018, the quality of SysMod contributions has been recognised through various awards. In 2018 in Chicago, Bhanwar Lal Puniya received the best poster award for his contribution to ‘Systems modelling of phenotypic plasticity of CD4+ T-cell differentiation’ (Puniya *et al.*, 2018). In 2019 in Basel, three best poster awards recognised the contributions of young researchers: (i) Ulrike Münzner, ‘A Mechanistically detailed model of the cell cycle in *Saccharomyces cerevisiae*’ (Münzner *et al.*, 2019), (ii) Kazunari Kaizu, ‘Whole Cell Simulation of Bacteria from Genomic Sequence,’ and (iii) José Américo N. Leva F. de Freitas, ‘Modelling gene regulatory networks in oncogene-induced senescence.’ In 2020, the best three virtual posters were awarded to (i) Chaitra Sarathy, ‘Identifying characteristic features of metabolic states using Genome-Scale Metabolic Models’ (Sarathy *et al.*, 2020), (ii) Narasimhan Balakrishnan, ‘Model reduction and optimal control for multicellular biological oscillator systems,’ and (iii) Aurélien Pélissier, ‘Computational model reveals a stochastic mechanism behind germinal centre clonal bursts’ (Pelissier *et al.*, 2020).

## 5 Related events and partnerships

In addition to the annual meeting, SysMod partners with other modelling communities to provide opportunities to engage in scientifically relevant events throughout the year. For example, during the [BC]^2^ conference in Basel in 2019, SysMod, in partnership with the Consortium for Logical Models and Tools (CoLoMoTo, colomoto.org; (Naldi *et al.*, 2015), organised a workshop focused on developing guidelines and best practices for annotation and curation of logical models (Niarakis et al., 2021). During the ECCB in 2020, two additional SysMod-related events took place: (i) the hands-on tutorial on modelling software tools (‘Computational modelling of cellular processes: regulatory versus metabolic systems’) and (ii) the workshop on ‘Advances in computational modelling of cellular processes and high-performance computing’ with the two SysMod coordinators, María Rodríguez Martínez and Jonathan R. Karr, as keynote speakers, and one SysMod coordinator, Anna Niarakis, as a co-organiser. Additional public outreach activities include the monthly Online Cell Modelling Seminar organised by the Center for Reproducible Biomedical Modelling (https://reproduciblebiomodels .org/dissemination-training/seminar/), the hackathons and conferences on Integrative Collaborative modelling in systems Medicine (INCOME) organised by Jan Hasenauer, Wolfgang Müller and Olaf Wolkenhauer (www.integrative-pathway-models.de), and the COVID-19 disease map initiative (Ostaszewski *et al.*, 2020a,b) with its weekly online presentation series.

## 6 Conclusion

SysMod has grown to a vital element of the ISMB/ECCB, complementing an already wide range of bioinformatics topics with systems biology modelling. This integration is only possible thanks to a remarkably agile computational and systems biology community that presents and actively proposes and discusses the latest developments of the field. SysMod provides a unique platform for a wide range of related activities, through which its members benefit from a regular exchange.

The fundamental principle that brings together the SysMod community is the increasing interest in *mechanistic computational models* as the key to gain new *knowledge* about biological processes. The SysMod community focuses on models that explicitly include the principles of natural sciences, such as biophysics and thermodynamics, interaction mechanisms or spatiotemporal dynamics. Models are developed as computational tools that incorporate a wide range of quantitative data to predict and understand how biological systems function. Driven by this core value, SysMod regularly fosters discussion among experts from various fields who contribute to transforming biology from a qualitative and descriptive scientific discipline to a quantitative science based on engineering and computational analysis. As such, SysMod has contributed to the development of systems biology as a young field. Regarding to global challenges such as the COVID-19 pandemic or climate change, SysMod, with its virtual and personal conferences and forums, paves the way for the establishments of new forms of friendly and cooperative interactions, which will determine the future of our global networking.

## Notes

Correspondence should be addressed to all of the SysMod coordinators. sysmod-coord@googlegroups.com
